# A Gateway-Based System for Fast Evaluation of Protein-Protein Interactions in Bacteria

**DOI:** 10.1371/journal.pone.0123646

**Published:** 2015-04-09

**Authors:** Thorsten Wille, Britta Barlag, Vladimir Jakovljevic, Michael Hensel, Victor Sourjik, Roman G. Gerlach

**Affiliations:** 1 Junior Research Group 3, Robert Koch-Institute, Wernigerode Branch, Wernigerode, Germany; 2 Division of Microbiology, School of Biology/Chemistry, University of Osnabrück, Osnabrück, Germany; 3 Center for Molecular Biology at the University of Heidelberg (ZMBH), DKFZ (German Cancer Research Center) -ZMBH Alliance, Heidelberg, Germany; 4 Max Planck Institute for Terrestrial Microbiology & LOEWE (state offensive for the development of scientific and economic excellence) Center for Synthetic Microbiology (SYNMIKRO), Marburg, Germany; Deutsches Krebsforschungszentrum, GERMANY

## Abstract

Protein-protein interactions are important layers of regulation in all kingdoms of life. Identification and characterization of these interactions is one challenging task of the post-genomic era and crucial for understanding of molecular processes within a cell. Several methods have been successfully employed during the past decades to identify protein-protein interactions in bacteria, but most of them include tedious and time-consuming manipulations of DNA. In contrast, the MultiSite Gateway system is a fast tool for transfer of multiple DNA fragments between plasmids enabling simultaneous and site directed cloning of up to four fragments into one construct. Here we developed a new set of Gateway vectors including custom made entry vectors and modular Destination vectors for studying protein-protein interactions via Fluorescence Resonance Energy Transfer (FRET), Bacterial two Hybrid (B2H) and split *Gaussia* luciferase (Gluc), as well as for fusions with SNAP-tag and HaloTag for dual-color super-resolution microscopy. As proof of principle, we characterized the interaction between the *Salmonella* effector SipA and its chaperone InvB via split Gluc and B2H approach. The suitability for FRET analysis as well as functionality of fusions with SNAP- and HaloTag could be demonstrated by studying the transient interaction between chemotaxis response regulator CheY and its phosphatase CheZ.

## Introduction

The availability of thousands of whole genome sequences of many bacterial species represents a tremendous source of information. However, there are still very limited options to deduce cellular functions based on interactions of the various gene products from these data. Therefore biochemical and functional characterization of proteins including interactions with their peers is a challenging task of the post genomic era. One crucial and time-consuming step in this process is the cloning of a gene of interest (GOI) into one or more specific vectors. To address this problem a number of elegant cloning systems suitable for simultaneous and parallel cloning of multiple genes have been developed which meet the demands for high-throughput analysis of large open reading frame (ORF) collections. Examples of these methods involve strategies like site-specific recombination [1], topoisomerase-mediated strand transfer [2] or *in vivo* annealing of single stranded overhangs [3]. Another technique is the Gateway recombinational cloning system [4]. In contrast to the other systems, which are limited for example by the requirements of special hosts, selection schemes or the vector properties, Gateway cloning overcomes most of these limitations. The Gateway cloning technique is based on the site-specific recombination properties of phage λ facilitating the integration of its DNA in the *E*. *coli* chromosome and the switch between lytic and lysogenic pathways [5]. The recombination occurs between specific recombination sequences termed *att*-sites. The Gateway system includes two recombination events called BP and LR reaction. Both reactions are *in vitro* versions of the integration and excision processes during the lytic and lysogenic pathways of phage λ. In case of the BP reaction, variants of the bacterial *att*B-site recombine with versions of the phage *att*P-site resulting in *att*L- and *att*R-sites. It is noteworthy that *att*-sites recombine not only efficient but also very specific with each other. For example *att*B1 only reacts with *att*P1 or *att*B2 only with *att*P2, insuring directional cloning of the DNA fragments. In Gateway systems, this step is used to integrate the GOI in a Donor vector to create an Entry clone, from which the GOI can be transferred via LR reaction to a myriad of Destination vectors tailored for individual applications. In addition to their wide application in the field of mammalian cells, invertebrates, plants, yeasts and fungi [6], flexible Gateway-based vector systems were also developed for use in bacterial hosts [7–9].

The expansion to four unique *att* recombination sites in the MultiSite Gateway system enabled the generation of complex constructs by the exchange of multiple DNA fragments between up to four plasmids in a single reaction [10]. Vectors using this approach are available for mammalian cells [6], plants [11] and also for Gram-positive bacteria [12]. Here we adapted this system for Gram-negative bacteria to study protein-protein interactions using reporter gene fusions. A new set of MultiSite Gateway vectors including custom-made Entry vectors for cloning of genes of interest and modular Destination vectors was constructed. These application-specific vectors can be used for fluorescence- and super-resolution microscopy, Fluorescence Resonance Energy Transfer (FRET), Bacterial two Hybrid (B2H) and split *Gaussia* luciferase (Gluc) assays. As proof of principle, we evaluated our system by investigating well-known interactions between the *Salmonella* Pathogenicity Island 1 (SPI1) Type III Secretion System (T3SS) effector SipA and its chaperone InvB as well as between the chemotaxis response regulator CheY and its phosphatase CheZ.

## Materials and Methods

### Bacterial strains, oligonucleotides, plasmids and cloning

Oligonucleotides used for cloning and mutagenesis were purchased HPLC-purified from Biomers (Ulm, Germany) and can be found in S1 Table. **Construction of knockout strains:** All gene knockouts were generated using Red recombination as described before [13]. Briefly, primers CheY-Red13-Del-For and CheY-Red13-Del-Rev or CheZ-Del-for and CheZ-Del-rev were used to amplify a kanamycin resistance cassette from pKD13 or pKD4 which were subsequently integrated within the chromosome to delete *cheY* or *cheZ*, respectively. The kanamycin resistance cassettes were removed in strains MvP1212 and WRG105 using transient expression of FLP recombinase from pCP20. Strain WRG106 was constructed by transferring the *cheZ*::*aph* cassette into MvP1212 by means of phage P22 HT105/1 *int-201* transduction. All strains used are listed in Table 1. The following cloning procedures were used for plasmid construction. **Destination vectors:** A DNA fragment containing *att*B1 and *att*B4 recombination sites, multiple restriction endonuclease recognition sites and the transcription termination sequence of the *rrnB* operon [14] was synthesized (Life Technologies, Regensburg, Germany) and cloned into pBluescript II SK+ (Stratagene) via KpnI/NotI yielding pWRG201. The *tetA*/*tetR*-promoter was amplified from pST98-AS [15] using primers Acc65I-SmiI-Pro-tet-for and Pro-tet-EcoRV-rev for cloning into pWRG201 via KpnI/EcoRV. The resulting vector pWRG239 was digested with KpnI/NotI for cloning the synthetic construct together with P_*tetA*_ into pWSK29. The resulting vector pWRG392 was used in a Gateway BP-reaction together with pDONR 221 (Life Technologies) yielding pWRG512. The reporter genes *syfp*2, *t18*, *gluc* ^M43L^_M18–105_ (*gluc*105) and *haloTag* were amplified using primer pairs BglII-XFP-for/XFP-NotI-rev, BglII-T18-for/T18-NotI-rev, BglII-SplitGLuc1-N-for/SplitGLuc1-N-NotI-rev and BglII-L14-HaloTag-for/HaloTag-NotI-rev from template vectors pSYFP2-C1 [16]; pUT18 [17], pMA-GLuc^M43L-M110L^ (synthetic construct, Life Technologies, Regensburg, Germany) and pHTC HaloTag CMV-neo (Promega). PCR products were digested with BglII/NotI and cloned into pWRG512, yielding vectors pWRG-FRET-DEST-C, pWRG-B2H-DEST-C, pWRG-GLUC-DEST-C and pWRG-HALO-DEST-C. For N-terminal reporter gene fusions *t25* was amplified from template vector pKT25 [17] using primer pair NaeI-RBS-T25-for/T25-NaeI-rev. The PCR product was digested with NaeI and cloned into pWRG-B2H-DEST-C, resulting in vector pWRG-B2H-DEST-N. **Entry clones:** Primers attB4r-for and attB3r-rev were used to amplify a 293 bp fragment with the synthetic construct as template. The PCR product was used in a Gateway BP-reaction together with pDONR 221 P4r-P3r to generate the Entry vector pWRG258. The reporter genes *scfp3a*, *t25*, *gluc* ^M110L^_106–185_ (*gluc*_106_) and *snap-Tag* were amplified with primer pairs HindIII-XFP-for/XFP-XhoI-rev, HindIII-T25-for/T25-XhoI-rev, HindIII-SplitGLuc1-C-for/SplitGLuc1-C-XhoI-rev and HindIII-L14-SnapTag-for/SnapTag-XhoI-rev, respectively from templates pSCFP3a-C1 [16], p25-N [18], pMA-T-GLuc^M43L-M110L^ or a synthetic SNAP-Tag construct (Life Technologies). After digestion with HindIII/XhoI the fragments were cloned into pWRG258, yielding plasmids pWRG-FRET-ENTR-C, pWRG-B2H-ENTR-C, pWRG-GLUC-ENTR-C and pWRG-SNAP-ENTR-C for C-terminal reporter gene fusions. For N-terminal reporter gene fusions *t18* was amplified from template pUT18C [17] using primer pair PmlI-RBS-T18-for/T18-PmlI-rev, digested with NaeI or PmlI and cloned into pWRG-B2H-ENTR-C, resulting in pWRG-B2H-ENTR-N. EcoRV and NotI restriction recognition sites were deleted form pDONR 221 P1-P4 and pDONR 221 P3-P2 via EcoRV/NotI restriction, Klenow-fill in and re-ligation, yielding pWRG175 and pWRG176, respectively. The primer pairs B1-B4-for/B1-B4-rev and B3-B2-for/B3-B2-rev were used to amplify a 87 bp, 84 bp, 98 bp and a 97 bp DNA fragment from the synthetic oligonucleotides E-PCR1_B1-B4, E-PCR2_B3-B2, attB1-entry-n-term-tag-attB4 and attB3-entry-n-term-tag-attB2. Performing a BP-Reaktion the PCR products were recombined with the specific Donor vectors pWRG175 and pWRG176, to create the Entry-vectors pWRG179, pWRG180, pWRG583 and pWRG584. The *ccd*B-CmR selection cassette was amplified from pDONR 221 using primers EcoRV-CmR-ccdB-for and CmR-ccdB-NotI-rev and cloned into pWRG179, pWRG180, pWRG583 and pWRG584, yielding pWRG-ENTR-C1, pWRG-ENTR-C2, pWRG-ENTR-N1 and pWRG-ENTR-N2. An overview of all plasmids is given in S2 Table.

**Table 1:** Strains used in this study.

Strain designation	Relevant characteristics	Source or Reference
NCTC 12023	*S*. Typhimurium wild-type, isogenic to ATCC 14028	NCTC, Colindale, UK
M913	*S*. Typhimurium SL1344 *fliGHI*::Tn*10* Tet^r^	[19]
MvP103	*S*. Typhimurium NCTC 12023 SPI2-negative, *sseC*::*aphT* Kan^r^	[20]
MvP1212	*S*. Typhimurium NCTC 12023 *cheY* FRT	This work
WRG105	*S*. Typhimurium NCTC 12023 *cheZ* FRT	This work
WRG106	*S*. Typhimurium NCTC 12023 *cheY* FRT; *cheZ*::*aph*; Kan^r^	This work
BTH101	*E*. *coli* F_ *cya*-99 *araD139 galE15 galK16 rpsL1* (Strr) *hsdR2 mcrA1 mcrB1*	[21]
VS104	*E*. *coli* Δ[*cheY*-*cheZ*)	[22]
One Shot Mach1-T1^R^	*E*. *coli* F- φ80(*lacZ*)ΔM15 Δ*lac*X74 *hsd*R(rK-mK+) Δ*rec*A1398 *end*A1 *ton*A	Life Technologies
One Shot *ccd*B Survival 2 T1^R^	*E*. *coli* F^-^ *mcr*A Δ*(mrr*-*hsd*RMS-*mcr*BC) Φ80*lac*ZΔM15 Δ*lac*X74 *rec*A1 *ara*Δ139 Δ(*ara*-*leu*)7697 *gal*U *gal*K *rps*L (Str^r^) *end*A1 *nup*G *fhu*A::IS2	Life Technologies

### Recombination reaction

Gateway BP- and LR reactions were carried out according to manufacturer's instructions (Gateway technology manual, Life Technologies). In short: For BP reactions the (*att*B) PCR product (15–150 ng, 1–7 μl) was mixed with the appropriate (*att*P) pDONR vector (supercoiled, 150 ng*μl^-1^; 1 μl) together with 1X TE Buffer, pH 8.0 to a final volume of 8 μl. Afterwards 2 μl of BP-clonase II enzyme mix (Life Technologies, Darmstadt, Germany) was added. The reaction was mixed briefly and incubated at 25°C for 1 hour. Subsequently, 1 μl of 2 μg*μl^-1^ Proteinase K solution was added and incubated at 37°C for 10 min. 2 μl of the reaction was transformed into One Shot Mach1 T1^R^ *E*. *coli* and selected for kanamycin resistant Entry clones. For MultiSite Gateway Pro LR recombination reactions three Entry clones (supercoiled, 10 fmoles each; 1–7 μl) were mixed with one Destination vector (supercoiled, 20 fmoles 1 μl) together with 1X TE Buffer, pH 8.0 to a final volume of 8 μl. Afterwards 2 μl of LR-clonase II Plus enzyme mix was added. The reaction was mixed briefly and incubated at 25°C for 16–24 hours. One μl of 2 μg*μl^-1^ Proteinase K (Life Technologies) solution was added and incubated at 37°C for 10 min. One Shot Mach1 T1^R^ *E*. *coli* were transformed using 2 μl of this reaction and resulting Expression clones were selected with carbenicillin.

### Bacterial two hybrid assay

*E*. *coli* reporter strain BTH101 [21] was freshly transformed with a Gateway Expression clone containing two translational fusions to the T18 or T25 fragments derived from *Bordetella pertussis* CyaA, respectively. After transformation, three μl of bacterial suspensions were spotted onto LB plates as described before [23].

### Measuring Gluc activity

Luciferase activity of split Gluc constructs was determined as described before [23].

### Motility assay

As an indicator of a functional chemotaxis system, the motility of different *Salmonella* strains was assessed on swarm plates. Briefly, a small amount (0.2 μl) of bacterial overnight cultures was applied onto the center of a semisolid agar plate (LB with 5 g*l^-1^ NaCl, 0.5% agar, 0.5% glucose) supplemented with varying amounts of anhydrotetracycline (AHT, Fluka 37919) as indicated. After incubation for 6–8 h at 34°C, the diameters of the swarm colonies were measured and the plates were photographed.

### Microscopy and flow FRET analyses

For localization of chemotaxis proteins or FRET measurements *S*. Typhimurium MvP103 (Δ*sseC*) and *E*. *coli* VS104 [Δ(*cheY-cheZ*)] carrying plasmid pWRG415, or *S*. Typhimurium NCTC 12023 carrying plasmid pWRG602 were grown in tryptone broth (TB) with ampicillin (100 μg*ml^-1^) at 30°C. Overnight cultures were diluted 1:100 in TB containing 50 ng*ml^-1^ AHT and were allowed to grow at 34°C in a rotary shaker until an OD_600_ = 0.45–0.5 was reached. Cells were collected by centrifugation (4°C, 4,000 rpm, 15 min.) and washed twice with tethering buffer (10 mM KPO_4,_ 0.1 mM EDTA, 1 μM methionine, 10 mM lactic acid, 67 mM NaCl, pH 7.0) before being attached to poly-L-lysine coated coverslips [24]. Microscopy of living bacteria was done using an epifluorescence microscope (Nikon Eclipse Ti) with a 100x 1.40 numerical aperture (NA) objective and appropriate CFP- and YFP filter sets. Fluorescence images of the individual channels were pseudo-colored and merged using ImageJ (http://imagej.nih.gov/ij/). To visualize covariance as an indicator for complex formation the product of the difference from the mean (PDM) was calculated and plotted using Image Correlation Analysis (ICA) by Stanley's ICA plugin for ImageJ [25]. For flow FRET analyses, bacteria-coated coverslips (see above) were placed in a flow cell [26] which was kept under constant flow of tethering buffer (0.5 ml*min^-1^) using a syringe pump (Harvard Apparatus). The flow was used to add and remove attractant α-methyl-DL-aspartic acid (MeAsp, Sigma-Aldrich M6001). Flow FRET measurements were carried out on a fluorescence microscope derived from the one described before [27]. The set-up is based on an Olympus IX81 inverted fluorescence microscope equipped with a 60x UPlanFLN 0.9 NA objective.

### dSTORM superresolution microscopy

Cells were grown as described before. For the last 45 min of subculture fluorescence ligands were added. CheZ-HaloTag was stained with 150 nM HaloTag ligand (HTL) coupled to Atto655 (self-synthesized) and CheY-SNAP-tag fusion protein was stained using 30 nM TMR-Star (NEB). Cells were collected by centrifugation (4°C, 4,000 rpm, 15 min.) and washed twice with tethering buffer and immobilized on agarose slides. TIRF Microscopy was performed using an inverted Olympus IX71 microscope equipped with a motorized quad-line total internal reflection (TIR) illumination condenser (Olympus), with 488 nm (250 mW), 568 nm (150 mW) and 647 nm (250 mW) lasers (Olympus) as well as a back-illuminated electron multiplied (EM) CCD camera (Andor iXon Ultra 897). A 150x 1.45 NA objective (UAPON, Olympus) was used for TIR-illumination. The excitation beam was reflected into the objective by a quad-line dichroic beamsplitter for reflection at 405 nm, 488 nm, 568 nm and 647 nm (Di01 R405/488/561/647, Semrock). 500 frames were recorded with an exposure time of 31 ms for 561 nm and 640 nm laser and laser power of 5 mW (objective output). Localization of single molecules was carried out using a modulated MTT version [28–30]. Colocalization analysis was done using the Matlab-based Slimfast software with a distance threshold of 100 nm [31].

## Results

**Fig 1:**
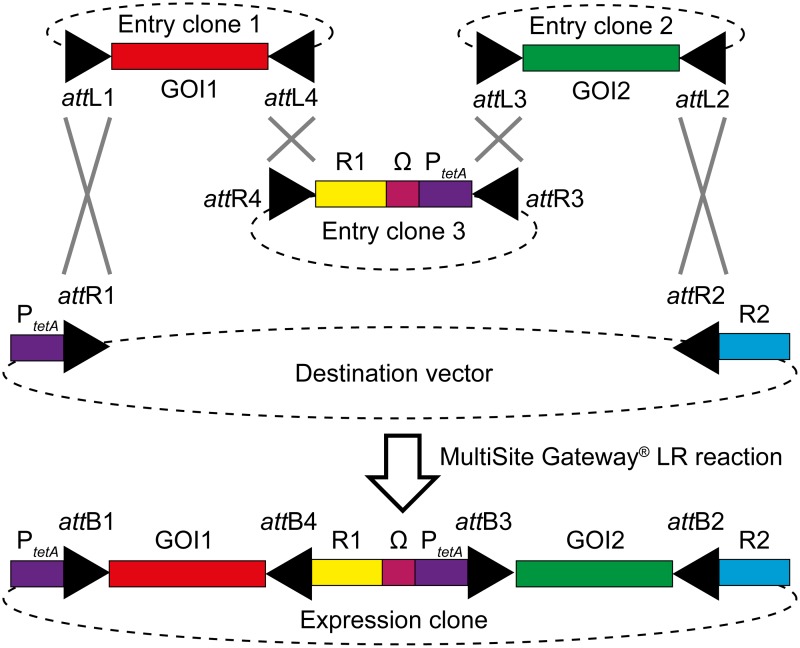
Adaptation of the MultiSite Gateway Pro 3-fragment recombination for use in bacteria. Two Entry clones containing each a gene of interest (GOI) recombine (crossed grey lines) together with a third Entry clone and one Destination vector harboring each a reporter gene (R1/2) to create an Expression clone. The Expression clone contains three DNA elements donated from the three Entry clones and enables expression of the GOI1-R1 and GOI2-R2 fusion genes from tetracycline-inducible promoters (P_*tetA*_). Transcription of fusion genes is stopped by a termination sequence derived from that of the *rrnB* operon (Ω).

Our system is based on a MultiSite Gateway three fragment recombination reaction (overview: Fig 1). Three Entry clones recombine in one step into a Destination vector yielding the desired Expression clone. Two of the three Entry clones encode the GOIs, the third Entry clone, as well as the Destination vector encode promoters and reporter genes for expression and generation of fusion proteins. The final Expression clone encodes two GOIs fused to the desired reporter genes with each fusion construct under control of a tetracycline-inducible promoter (P_*tetA*_).

### Construction of Entry clones encoding the GOIs

**Fig 2:**
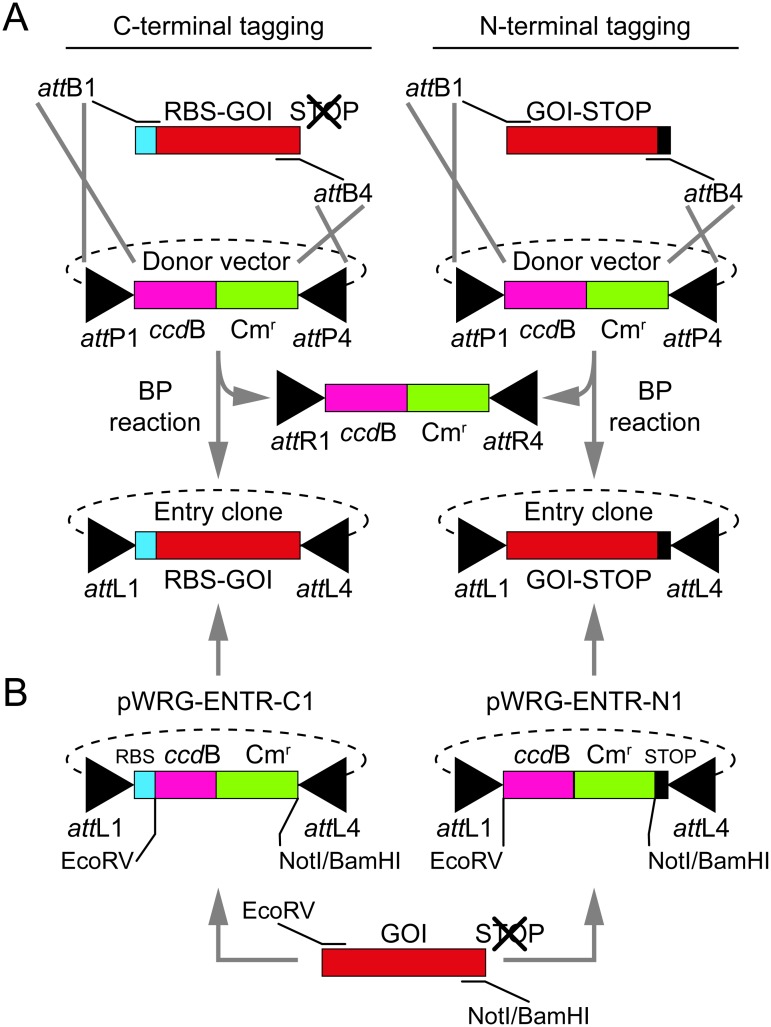
Generation of Entry clones. (A) Generation of Entry clones via BP reaction. The genes of interest (GOI, red) were amplified with flanking *att*B-sites and four terminal G’s (not shown). Depending on whether the GOIs should be fused N- or C-terminally to a reporter gene, the genes were amplified together with a ribosome binding site (RBS, turquoise) and no stop-codon (left) or without RBS and with stop-codon (right, black rectangle). Afterwards, the PCR products were recombined in a BP reaction with an appropriate pDONR vector yielding the desired Entry clones. (B) Generation of Entry clones via ‘classical’ cloning. The GOI is amplified with primers containing the flanking restriction enzyme recognition sites as indicated (bottom) and cloned in exchange with a *ccd*B-Cm^r^ selection cassette into a custom-made Entry vector for C- (pWRG-ENTR-C1, left) or N-terminal (pWRG-ENTR-N1, right) reporter gene fusions, resulting in the desired Entry clone. Only the generation of *att*L1-*att*L4 Entry clones is shown. Construction of *att*L3-*att*L2 Entry clones follows the same principles, but alternative Entry- and Donor vectors and suitable primers have to be used (not shown).

There are two ways of creating Entry clones harboring the GOIs. The first one is a BP reaction. Therefore the GOI must be amplified via PCR using primers with extensions including *att*B-sites and four terminal guanines (Fig 2A). If a C-terminal reporter gene fusion should be generated, the GOI must be amplified together with a ribosome binding site (RBS) and no stop codon (Fig 2A, left). For N-terminal fusions, the RBS is provided by the reporter but the stop codon needs to be included when amplifying the GOI (Fig 2A, right). Afterwards, the PCR product is recombined with an appropriate pDONR vector encoding the complementary *att*P-sites flanking a *ccd*B-Cm^r^ selection cassette. The products of the BP reaction are the Entry clone encoding the GOI flanked by now *att*L-sites and a by-product consisting of the selection cassette and terminal *att*R-sites.

The second way is "classical" cloning of the GOI via restriction and ligation. For that purpose dedicated Entry vectors for N- and C-terminal reporter gene fusions were constructed (Fig 2B). All of these Entry vectors contain a *ccd*B*-*Cm^r^ selection cassette flanked by *att*L-sites. This selection cassette is depleted in exchange for the GOI via the flanking EcoRV and NotI or BamHI restriction enzyme recognition sites. The Entry vectors for C-terminal reporter gene fusions (pWRG-ENTR-C1/2) have an optimized RBS in front of the selection cassette. In contrast Entry vectors for N-terminal reporter gene fusions (pWRG-ENTR-N1/2) carry stop codons after the BamHI and NotI restriction recognition sequence (Fig 2B, right). The advantage of this Entry clone architecture is the possibility to amplify the GOIs for N- and C-terminal reporter gene fusions with the same primers excluding the native stop codon. The Entry clones resulting from “classical” cloning contain the additional restriction enzyme recognition sites compared to those originating from a BP reaction. The integrity of the inserted GOI can be checked by sequencing using primers ENTR-Seq-for and ENTR/EXPR-Seq-rev (S1 Table).

### Construction of the Destination vector and third Entry clone

Construction of the Destination vector pWRG512 was based on pWSK29, a low-copy vector with an f1 origin of replication [32]. This vector was modified with an *att*R1-*ccd*B-Cm^r^-*att*R2 recombination locus transforming it into a Destination vector. Furthermore, a tetracycline-inducible promoter (*tetR*-P_*tetA*_) was integrated upstream of the recombination locus. The third Entry clone (pWRG258) was generated via BP reaction of pDONR P4r-P3r with a synthetic *att*B4-*rrnB*-terminator-P_*tetA*_-*att*B3 construct (Life Technologies, Regensburg, Germany). The resulting Entry clone encodes an *rrnB* transcription termination sequence together with P_*tetA*_ flanked by *att*R4-*att*R3 sites.

**Fig 3:**
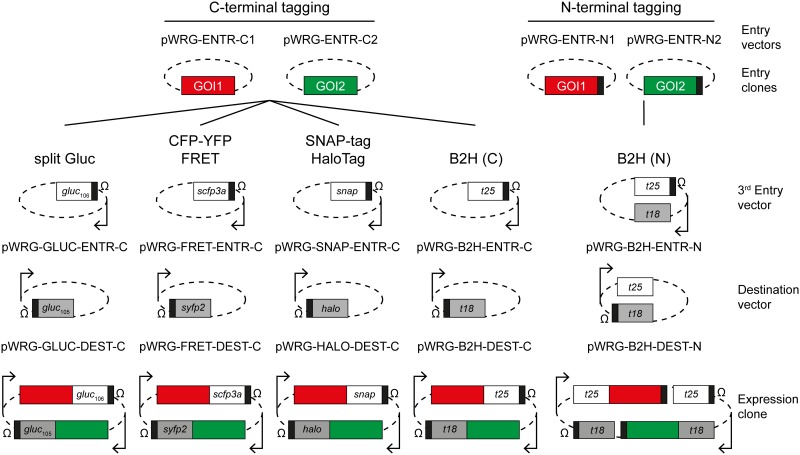
Overview of the system and plasmid combinations. Two Entry vectors suitable for C-terminal (left) or N-terminal (right) tagging are modified with genes of interest (GOI1 = red, GOI2 = green). The two resulting Entry clones are combined in a MultiSite Gateway LR reaction with an application-specific 3^rd^ Entry clone and Destination vector (below) resulting in the desired Expression clone (bottom). Plasmids pWRG-B2H-ENTR-N and pWRG-B2H-DEST-N allow for N-terminal tagging in bacterial two hybrid (B2H) applications if used together with compatible pWRG-ENTR-N1/2-derived Entry clones (right). Boxes represent the genes or gene fragments as indicated (*gluc*_105_ = Gluc_M18–105_, *gluc*_106_ = Gluc_106–185_, *scfp3a* = Super CFP 3a, *syfp2* = Super YFP 2, *snap* = SNAP-tag, *halo* = HaloTag 7, *t18* = T18 fragment of CyaA from *B*. *pertussis*, *t25* = T25 fragment of CyaA from *B*. *pertussis*), black rectangles symbolize stop-codons, tetracycline-inducible promoters are depicted as arrows and Ω stand for *rrnB*-derived terminators. Resistance cassettes, *att*R- and *att*L sites as well as by-products of the reactions are not shown.

To evaluate the functionality of the customized MultiSite Gateway vectors, a LR reaction was performed with the two Entry vectors for C-terminal reporter gene fusions (pWRG-ENTR-C1/2), the third Entry clone (pWRG258) and the Destination vector (pWRG512). Transformation of *E*. *coli* Mach1 T1 chemically competent cells with the LR reaction mix yielded 81 ampicillin resistant colony forming units (cfu). Further analysis by colony PCR and control digest showed that all seven clones screened had the expected insertion of DNA fragments donated from the Entry clones. Sequencing of three clones using primers EXPR-Seq-for and ENTR/EXPR-Seq-rev (S1 Table) confirmed the integrity of the two 29 bp insertions originating from pWRG-ENTR-C1/2 as well as of the 242 bp insertion from pWRG258. In a next step we modified plasmids pWRG512 and pWRG258 with various reporter genes for investigating protein-protein interactions via B2H, FRET, split Gluc and as well as for fusions with SNAP-tag and HaloTag. An overview of the system is given in Fig 3. To test the resulting MultiSite Gateway vectors, we used well-known protein-protein interactions of *Salmonella enterica* serovar Typhimurium (*S*. Typhimurium). One example is the interaction between the *Salmonella* Pathogenicity Island 1 (SPI1) Type III Secretion System (T3SS) effector SipA and its cognate chaperone InvB. Binding of the chaperone not only keeps the effector in an export-competent, at least partially unfolded state, but also enables the interaction with the T3SS. Both activities are essential for the subsequent effector translocation process [33]. Previous studies identified the chaperone binding domain (CBD) of SipA within the first 47 N-terminal amino acids and SipA variants lacking this part are unable to form stable complexes with InvB *in vitro* and *in vivo* [23,34]. Here we created Entry clones for C-terminal reporter gene fusions encoding InvB, SipA as well as SipA^48–685^, a SipA variant lacking its first 47 amino acids, via “classical” cloning as starting point for B2H and split Gluc analysis (S2 Table).

### Bacterial two-hybrid assays

**Fig 4:**
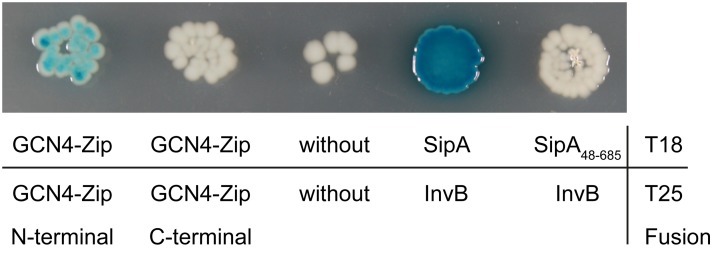
Gateway-based B2H assay for detection of chaperone-effector interaction. Expression clones encoding reporter gene fusions GCN4-Zip N-terminal (T25-GCN4-Zip/T18-GCN4-Zip), GCN4-Zip C-terminal (GCN4-Zip-T25/GCN4-Zip-T18), unfused T18 and T25 fragments (without), SipA-InvB (InvB-T25/SipA-T18) and SipA_48–685_-InvB (InvB-T25/SipA_48–685_-T18) were co-expressed in the *cyaA*-deficient *E*. *coli* B2H reporter strain BTH101. Blue colonies indicate protein-protein interaction through T18/T25 CyaA fragment complementation.

The B2H assay developed by Karimova *et al*. [17], is based on functional complementation between the two complementary fragments T18 and T25 of the catalytic domain of *Bordetella pertussis* adenylate cyclase CyaA. We cloned the *t18* and *t25* fragments into the Destination vector pWRG512 and into the third Entry clones for C- and N-terminal reporter gene fusions, yielding Destination vectors pWRG-B2H-DEST-C and pWRG-B2H-DEST-N and Entry vectors pWRG-B2H-ENTR-C and pWRG-B2H-ENTR-N. LR reactions with the two Entry clones encoding InvB (pWRG367) and SipA (pWRG368) or InvB and SipA^48–685^ (pWRG369) together with pWRG-B2H-DEST-C and pWRG-B2H-ENTR-C were performed. After transformation of *E*. *coli* Mach1 T1 with the LR reaction mixes, 50% and 37.5% of the resulting Expression clones pWRG448 and pWRG450 were shown to contain the expected insert using colony PCR (S3 Table). The leucine zipper motif of the yeast GCN4 transcriptional activation domain was already used as positive control for B2H by Karimova *et al*. [17]. We constructed in parallel Gateway vectors for N-terminal as well as C-terminal *t18*/*t25* gene fusions to this motif because N-terminal fusions were successfully used before [17]. LR reactions of these Entry clones together with Destination vector pWRG-B2H-DEST-C or pWRG-B2H-DEST-N and Entry vector pWRG-B2H-ENTR-C or pWRG-B2H-ENTR-N yielded 87.5% or 100% positive Expression clones pWRG482 and pWRG623 (S3 Table). As background control Entry vectors encoding *t18* and *t25* were generated and recombined with Destination vector pWRG512 and the third Entry vector pWRG258 yielding Expression clone pWRG534. We identified 43.8% correct clones of pWRG534 using colony PCR (S3 Table). Afterwards the generated Expression clones were transformed into the *cyaA*-deficient B2H *E*. *coli* reporter strain BTH101. Following transformation the interactions were analyzed on IPTG/X-Gal-containing agar plates (Fig 4). Co-expression of SipA-T18 and InvB-T25 led to the formation of blue colonies indicating an interaction between both fusion proteins as expected. In line with this, deletion of the SipA CBD in SipA^48–685^ abrogates the interaction with InvB, leading to white colonies similar to the background control. Interestingly, only N-terminal fusions of T18 and T25 to the leucine zipper motif resulted in functional complementation of the two adenylate cyclase fragments, while co-expression of C-terminal fusion proteins failed for fragment complementation (Fig 4).

### Split Gluc fragment complementation assays

**Fig 5:**
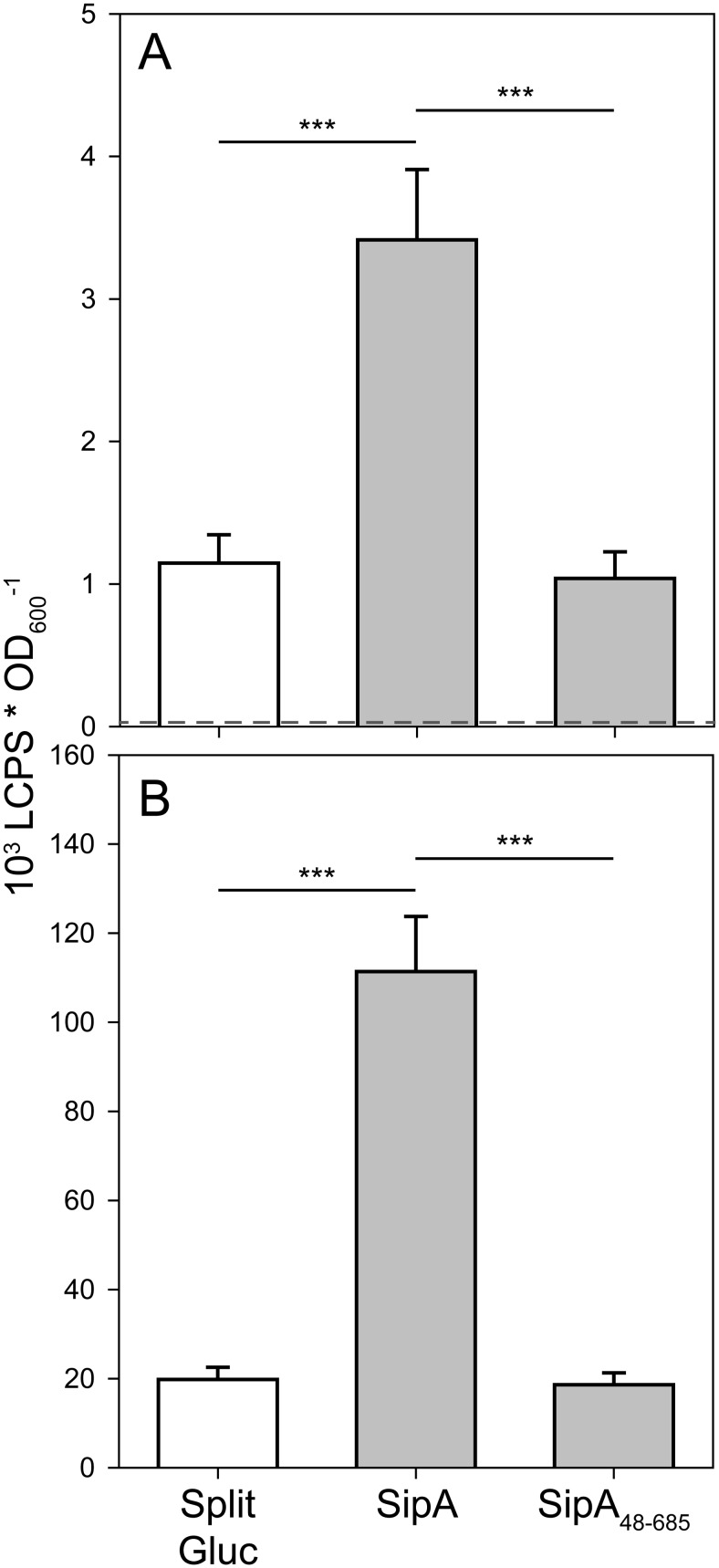
Gateway-based split Gluc protein complementation assay (PCA) for detection of chaperone-effector interaction. Expression clones encoding InvB fused to Gluc^M43L^_105_ and SipA or a SipA variant lacking its CDB (SipA_48–685_) fused to Gluc^M110L^_106_ or Gluc^M43L^_105_ and Gluc^M110L^_106_ alone (split Gluc) were transferred into *S*. Typhimurium and expression was induced with 50 ng*ml^-1^ AHT. Gluc activity was quantified from intact cells (A) and bacterial lysates (B). The dotted line in (A) represents the background luminescence determined with an empty vector control (pWSK29). Mean and standard deviation out of three independent experiments done in triplicate is shown. Statistical analysis by Student’s *t*-test was done by comparing individual strains as depicted: ***, *P* < 0.001.

Gluc is the smallest known coelenterazine (CTZ)-utilizing luciferase (~20 kDa) and generates over 1,000-fold higher luminescence intensities compared to native *Renilla reniformis* or *Firefly luciferase* [35]. Two split variants of Gluc were developed for use in protein complementation assays (PCA) to detect protein-protein interactions in living cells [36, 37]. To access the Gateway system for split Gluc analysis, Destination vector (pWRG-GLUC-DEST-C) as well as third Entry clone (pWRG-GLUC-ENTR-C) were constructed encoding the split Gluc fragments according to Remy and Michnick [34]. LR reaction with the Entry clones encoding InvB, SipA and SipA^48–685^ yielded Expression clones pWRG471 and pWRG473, respectively. As background control, Entry vectors encoding both split Gluc fragments were generated and recombined with Destination vector pWRG512 and the third Entry vector pWRG258 yielding Expression clone pWRG469. Screening of a subset of the colonies resulting from these LR reactions by colony PCR yielded between 37.5% and 75% correct clones (S3 Table). Subsequently, the Expression clones were transformed into *S*. Typhimurium and luminescence activity was quantified from whole cultures and bacterial lysates. Co-expression of full-length SipA together with InvB resulted in 6-fold and 3-fold higher Gluc activity in lysates and intact cells, respectively, compared to cells expressing SipA^48–685^ and InvB (Fig 5). Western blot analyses demonstrated that these differences in luminescence activity were not the results of different expression levels of the two SipA variants (S1 Fig).

### Using FRET to measure dynamic protein-protein interactions

In contrast to the B2H- and split Gluc approaches, FRET is qualified for investigating protein-protein interactions in real time *in vivo* making it particularly useful in cases when interactions are transient. In this regard, the dynamic interaction between the diffusible chemotaxis protein CheY and its phosphatase CheZ is one of the best studied examples. The cytoplasmic signaling protein CheY is phosphorylated in the absence of a chemotactic attractant or in the presence of a repellant by CheA which is bound to methyl-accepting chemotaxis protein (MCP) sensors. CheZ binds and dephosphorylates phosphorylated CheY (CheY~P) which subsequently reduces the half-life of CheY~P to only a few tenth of a second. This very labile and transient interaction was characterized before using FRET in combination with CFP- (cyan fluorescent protein) and YFP (yellow fluorescent protein) tagged CheY and CheZ proteins [22]. For the Gateway-based generation of these fusion genes, *cheY* and *cheZ* were amplified from *Salmonella* genomic DNA and cloned into the Entry vectors for C-terminal reporter gene fusions resulting in the Entry clones pWRG256 and pWRG257. After cloning the gene for super YFP 2 (*syfp2*) into the Destination vector pWRG512 and that for super CFP 3a (*scfp3a*) into the third Entry vector pWRG258, the resulting vectors pWRG-FRET-DEST-C and pWRG-FRET-ENTR-C were used to generate C-terminal reporter fusions. The LR reaction between the *cheY*, *cheZ* and *scfp3a* harboring Entry clones pWRG256, 257, 275 and pWRG-FRET-DEST-C resulted in the Expression clone pWRG415 encoding fusions of CheY to YFP and CheZ to CFP. The efficiency of the LR reaction was 87.5% (S3 Table). Functionality of the fusion proteins was analyzed by testing for complementation of the corresponding *S*. Typhimurium *cheYZ* double knock-out mutant WRG106 using a motility assay in swarm agar. S2 Fig shows that chemotactic motility of WRG106 was restored to about 86% of wild-type *S*. Typhimurium upon addition of 50 ng*ml^-1^ AHT which induces the expression of the CheY-YFP and CheZ-CFP fusion proteins from pWRG415. In the absence of AHT, WRG106 [pWRG415] showed no motility as observed for mutants deficient for flagella (*fliGHI*) or chemotaxis proteins (*cheY*, *cheZ*) (S2 Fig).

**Fig 6:**
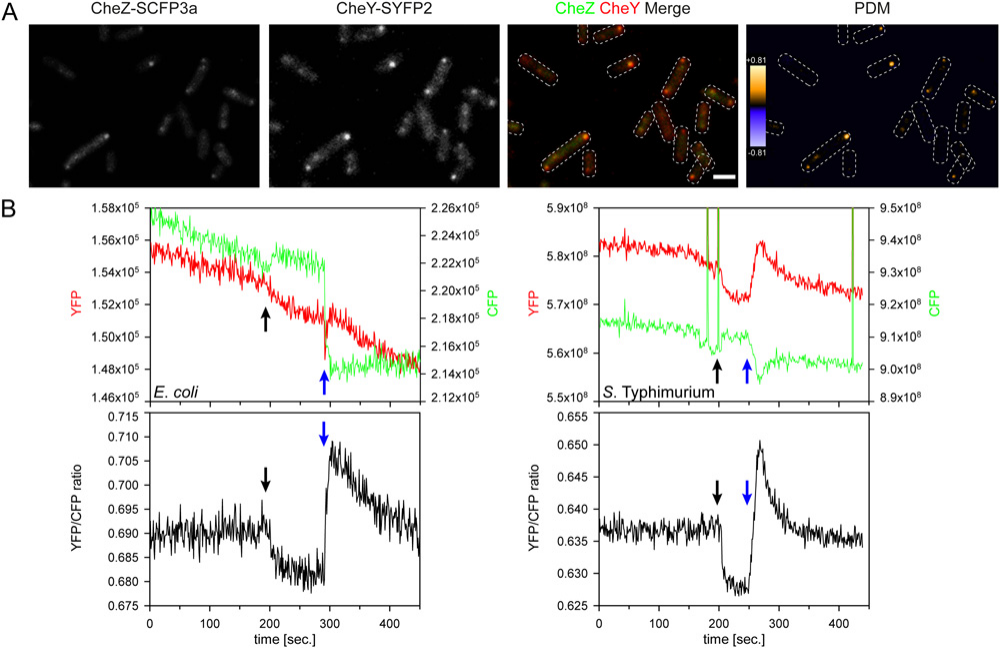
Localization and interaction of chemotaxis proteins CheY and CheZ. (A) Fluorescence microscopy of *S*. Typhimurium MvP103 (Δ*sseC*) cells co-expressing CheY-SYFP2 and CheZ-SCFP3a fusion proteins from Expression clone pWRG415. Signals from CFP- and YFP channels are shown separately as well as merged and pseudo-colored as indicated. Both fusion proteins formed intense clusters at the poles of the bacterial cells. The product of the difference from the mean (PDM) of both channels was calculated and plotted to show sites of complex formation (orange). The shapes of individual bacteria were illustrated by dashed white lines. Scale bar = 1 μm, Scale in PDM image = positive covariance (orange) and negative covariance (blue) (B) The same Expression clone as in (A) was used to detect the dynamics of the CheY-Z interaction by FRET in *E*. *coli* VS104 [Δ(*cheY-cheZ*)] (left) and *S*. Typhimurium MvP103 (right) cells. The fluorescence intensities of the CFP (green) and YFP (red) channels are depicted in the upper graphs. The YFP/CFP ratios calculated from the fluorescence intensities are shown below. Addition (black arrows) and removal (blue arrows) of the chemotactic attractant MeAsp resulted in a decrease (no interaction) or increase (interaction) of the YFP/CFP quotient, respectively.

Previous localization studies of CheY and CheZ in *E*. *coli*, performed by Sourjik and Berg [24] demonstrated that both proteins tend to cluster intensively at the poles as well as weaker laterally of the cell body. Here we could confirm this localization with the Gateway-derived CFP and YFP fusion proteins in *Salmonella* (Fig 6A). Furthermore, the two fluorescence channels showed positive covariance after calculation of the product of the difference from the mean (PDM) at the cell poles indicating complex formation at these subcellular sites (Fig 6A). Based on these results we investigated if our system is also suitable to analyze the inducible dynamic interaction between CheY~P and CheZ. Therefore *Salmonella* as well as *E*. *coli* cells expressing both fusion proteins were immobilized as dense monolayer on poly-L Lysine coated coverslips in a flow cell. Addition of the nonmetabolizable aspartate analog α-methyl-DL-aspartic acid (MeAsp) decreases kinase activity of CheA bound to MCPs resulting in lower levels of CheY~P [22]. Although MeAsp is bound by the aspartate receptor Tar/CheM only, other MCPs are involved in signal generation in a highly cooperative manner [38]. In line with these results addition of MeAsp by flow (black arrow, Fig 6B) led to a decrease in the YFP/CFP ratio due to a reduced interaction rate between CheY-YFP and CheZ-CFP in *E*. *coli* as well as *S*. Typhimurium (Fig 6B). Furthermore, removal of the attractant MeAsp by medium exchange (blue arrow, Fig 6B) resulted in an increase of the YFP/CFP ratio. In the absence of the attractant the higher cellular concentration of CheY~P resulted in a higher frequency of CheY~P-CheZ interaction as expected (Fig 6B).

### Dual-color super-resolution microscopy with SNAP-tag and HaloTag fluorescence ligands

**Fig 7:**
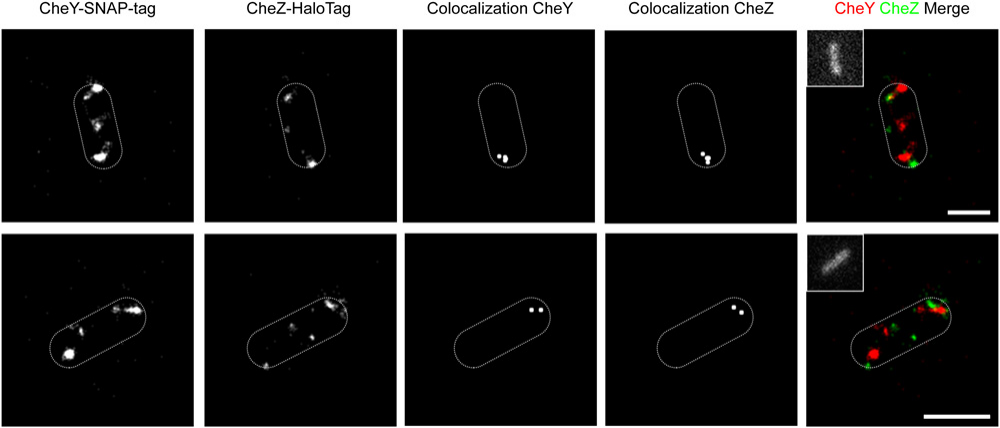
Subcellular localization of chemotaxis proteins CheY and CheZ. Two representative *S*. Typhimurium cells co-expressing fusions of CheY and CheZ to Halo- and SNAP-Tag from Expression clone pWRG602 are shown. Fusion proteins were labeled with Atto655-coupled HaloTag ligand and the SNAP-tag ligand TMR-Star. Subcellular localization of protein clusters was visualized using super-resolution microscopy (dSTORM). Colocalization of the labeled fusion proteins was calculated in separate images using the Matlab-based software Slimfast with a distance threshold of 100 nm. The insets show autofluorescence of the bacteria after excitation at 488 nm which was used to determine their shapes (dashed white lines). Scale bars = 1 μm.

The previously introduced constructs were always limited to one application. In contrast, the SNAP-tag [39] and HaloTag [40] systems enable covalent attachment of nearly any molecule (e.g. fluorophores, biotins or beads) to a protein of interest with the creation of a single fusion gene construct. The availability of optimized cell-permeable fluorescent ligands makes a combination of both systems very attractive for multicolor super-resolution microscopy [30]. To gain access to this application via our Gateway system, a Destination vector (pWRG-HALO-DEST-C), as well as a third Entry clone (pWRG-SNAP-ENTR-C) for C-terminal reporter gene fusions with *haloTag* and *snapTag* were constructed. A LR reaction was performed together with the *cheY* and *cheZ* harboring Entry clones pWRG256/7 which resulted in the Expression clone pWRG602. This Expression clone encodes fusions of CheY to HaloTag and CheZ to SNAP-tag. The efficiency of this LR reaction reached 75% (S3 Table). To demonstrate their functionality, the CheY-SNAP-tag and CheZ-HaloTag fusion proteins were co-expressed in *Salmonella* and the subcellular localization was studied with super-resolution microscopy [41]. Living *Salmonella* were treated with a membrane-permeable HaloTag ligand coupled to Atto655 and the SNAP-tag ligand TMR-Star. Subsequent analysis using direct stochastic optical reconstruction microscopy (dSTORM) with an optical resolution of ~20 nm revealed a dominant polar localization of CheY and CheZ (Fig 7). Colocalization of the labeled fusion proteins was calculated in separate images using the Matlab-based software Slimfast with a distance threshold of 100 nm. After that analysis interactions of both proteins were observed at the cell poles (Fig 7).

## Discussion

The Gateway cloning technology applied here offers a unique modular approach where the Entry clone can serve as a universal donor of the GOI for several downstream applications. With the design of custom, MultiSite Gateway-compatible Entry vectors we aimed at minimizing the cloning effort without sacrificing flexibility. Therefore, our vectors support not only insertion of the GOI via BP reaction but also ‘classical’ cloning of the GOIs for N- and C-terminal reporter gene fusions using the same PCR products. The negative selection scheme offered by the *ccdB*-*cat* cassette included in pWRG-ENTR-C1/2 and pWRG-ENTR-N1/2 makes ‘classical’ cloning of the GOI highly efficient with typically more than 90% positive clones. Once created, the GOI can be transferred easily from the Entry clone via LR reaction into our custom made Destination vectors for FRET, split Gluc and B2H analysis as well as for fusions with SNAP-tag and HaloTag. The availability of many ligands for SNAP-tag and HaloTag opens additional areas of application (e.g. affinity purification) making our system even more flexible. Besides these advantages it should also be noted that BP and LR reactions are based on conservative recombination meaning that there is no gain, loss or alteration in the nucleotide sequence which usually makes re-sequencing of the final Expression clones obsolete. Sequencing is often required to confirm constructs resulting from classical but also alternative cloning techniques involving PCR such as Gibson assembly [42]. Using this method to construct more complex vectors, e.g. for expression of two genes, requires multiple PCR products with larger overlaps and might exhibit lower efficiency (New England Biolabs). Gibson assembly can produce seamless links between the GOI and a reporter gene whereas in the Gateway system individual parts are always linked by *att*-sites. Linker sequences consisting of the ‘innocuous’ amino acids serine and glycine are an approved tool to minimize steric hindrances between two fusion partners. In contrast, the *att* sequence partially encodes for bulky amino acid residues which may interfere with protein folding and activity. Although we did not observe any negative impact of the *att* linker, the possibility of detrimental effects of these linkers as well as of the fused reporters or tags on protein function should be considered.

Previously an approach based on the recombination of three Entry clones with one Destination vector in a single MultiSite Gateway LR reaction was published for Gram-positive bacteria. This system allows for the generation of transcriptional reporter fusions by combining a promoter with a reporter and a terminator sequence [12]. Our system allows for the simultaneous generation of two translational reporter gene fusions using the same MultiSite Gateway approach in Gram-negative bacteria. This is in contrast to a previously published system which required the sequential insertion of two ORFs using a combination of Gateway/*att* and Cre/*loxP* recombination in *E*. *coli* [43]. To ensure equal expression of both fusion proteins, separate *tetA* promoters were included on the third Entry clone as well as on the Destination vector. The *tetR*-P_*tetA*_ repressor/promoter cassette was chosen because this promoter is tightly regulated and much less prone to all-or-none expression patterns [44] as observed with the widely used *araC*-P_BAD_ promoter [45]. Together with the pWSK29-derived low-copy-number backbone of the Expression clones, this allows for precise adjustment of expression levels to find a compromise between nearly endogenous (functional) levels, as assessed by complementing a corresponding knockout strain, and reporter signal.

The identification and further characterization of protein-protein interactions are important steps in understanding fundamental processes but also virulence functions of bacteria. The Gateway technology was able to facilitate the generation of affinity-tagged proteins for the identification of co-purified unknown interaction partners after affinity purification of complexes using mass spectrometry (APMS) [9]. In contrast our system offers different options to either confirm a putative interaction or for a detailed (*in vivo*) characterization of a known protein-protein interaction in bacterial cells. Hence functionality of our system for split Gluc and B2H analysis was shown by investigating the well-known interaction between the SPI1-T3SS effector protein SipA and its cognate secretion chaperone InvB. This interaction could be detected with both reporters and was depending on the presence of the SipA CBD. These results are in accordance with our previous study, where both fusion proteins were expressed from separate plasmids [23]. The interaction between SipA and InvB is thought to be stable upon binding of the chaperone-effector complex to the T3SS apparatus [34]. In contrast, interaction between chemotaxis protein CheY and its phosphatase CheZ is highly dynamic, with a half-life of a only few tenths of a second of CheY~P in presence of CheZ [22]. After successfully assessing more stable interactions between SPI4 proteins using our Gateway-based FRET vectors [46], we evaluated our system for flow FRET, which is one of the few methods qualified for investigation of protein-protein interactions in real time *in vivo*. In accordance with previous results [22] we could observe the inducible dynamic interaction between CheY and CheZ upon addition and removal of the chemotactic attractant MeAsp. Moreover, CFP and YFP fusion proteins expressed from the Gateway vector showed a predominant polar localization as observed previously [24]. For CheY this subcellular localization was also seen with photoactivated localization microscopy (PALM) reaching ~15 nm resolution [47]. Using dual-color dSTORM in combination with a Gateway-based co-expression vector allowed for parallel detection of CheY and CheZ in living cells at a comparable spatial resolution (~20 nm). Both proteins were predominantly found in polar clusters but, to a lower extent, also at the lateral sides of the bacteria. In the absence of repellant (or presence of attractant) CheY and CheZ show a rather low level of interaction [22]. Nevertheless, at the cell poles several events of CheYZ colocalization were observed under the conditions used for super-resolution microscopy.

In conclusion, our system provides a valuable tool for investigating protein-protein interactions in Gram-negative bacteria. The approach simplifies cloning steps and its modular nature enables evaluation of different methods and, if required, fast adaption to any other kind of fusion partners.

## Supporting Information

S1 Fig Western-Blot detecting Gluc fusion proteins in bacterial lysates. Samples were subjected to protein electrophoresis using Bis-Tris gradient gels. A polyclonal anti-Gluc antibody was used to detect SipA^1–685^-C-Gluc_106_ and SipA^48–685^-C-Gluc_106_. InvB-N-Gluc_105_ could not be detected by this antibody (upper panel). As a control, DnaK was detected using a monoclonal antibody (lower panel).
(PDF)

S2 Fig Functional analysis of CheY-YFP and CheZ-CFP fusion proteins. Functionality of the chemotaxis system was assessed using the swarming behavior of different *S*. Typhimurium strains on soft agar plates. (A) The swarm colony formation by *Salmonella* NCTC 12023 wild-type (WT) served as a positive control. Strain M913 (Δ*fliGHI*) is a nonmotile control and strains MvP1212 (Δ*cheY*) and WRG105 (Δ*cheZ*) do not show chemotaxis. (B) Swarm colony formation by strain WRG106 Δ*cheYZ* [pWRG415] expressing CheY-YFP and CheZ-CFP upon addition of different concentrations of the inductor anhydrotetracycline (AHT). Percentages indicate the diameter of the swarm colony in % of the WT control.
(PDF)

S1 Table Oligonucleotides used in this study. (DOCX)

S2 Table Plasmids used in this study. (DOCX)

S3 Table Efficiencies of LR reactions. (DOCX)
