# Supplementary material for: A Gateway-Based System for Fast Evaluation of Protein-Protein Interactions in Bacteria
Source: PLoS One. 2015 Apr 9;10(4):e0123646. doi: 10.1371/journal.pone.0123646 (PMC4391838; doi:10.1371/journal.pone.0123646)
Supplement: S1 Fig — Samples were subjected to protein electrophoresis using Bis-Tris gradient gels. A polyclonal anti-Gluc antibody was used to detect SipA1–685-C-Gluc106 and SipA48–685-C-Gluc106. InvB-N-Gluc105 could not be detected by this antibody (upper panel). As a control, DnaK was detected using a monoclonal antibody (lower panel). (PDF) [file pone.0123646.s001.pdf]

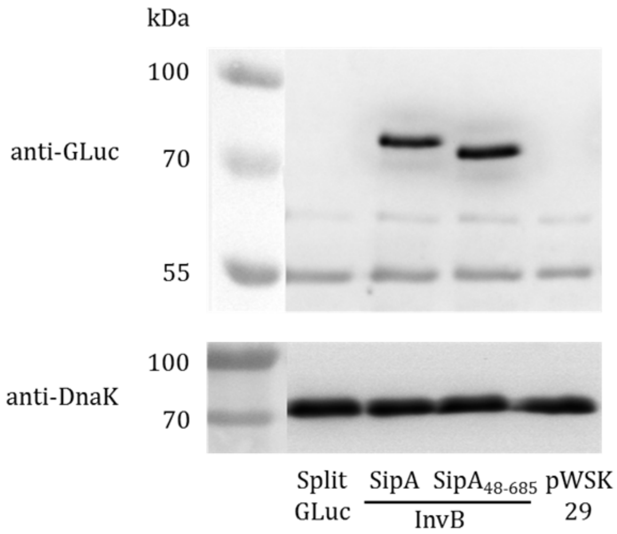

S1 Figure. Western-Blot detecting Gluc fusion proteins in bacterial lysates. Samples were subjected to protein electrophoresis using Bis-Tris gradient gels. A polyclonal anti-Gluc antibody was used to detect $SipA^{1\text{-}685}$-C-$Gluc_{106}$ and $SipA^{48\text{-}685}$-C-$Gluc_{106}$. InvB-N-$Gluc_{105}$ could not be detected by this antibody (upper panel). As control DnaK was detected with a monoclonal antibody (lower panel).
