# Supplementary material for: A Gateway-Based System for Fast Evaluation of Protein-Protein Interactions in Bacteria
Source: PLoS One. 2015 Apr 9;10(4):e0123646. doi: 10.1371/journal.pone.0123646 (PMC4391838; doi:10.1371/journal.pone.0123646)
Supplement: S2 Fig — Functionality of the chemotaxis system was assessed using the swarming behavior of different S. Typhimurium strains on soft agar plates. (A) The swarm colony formation by Salmonella NCTC 12023 wild-type (WT) served as a positive control. Strain M913 (ΔfliGHI) is a nonmotile control and strains MvP1212 (ΔcheY) and WRG105 (ΔcheZ) do not show chemotaxis. (B) Swarm colony formation by strain WRG106 ΔcheYZ [pWRG415] expressing CheY-YFP and CheZ-CFP upon addition of different concentrations of the inductor anhydrotetracycline (AHT). Percentages indicate the diameter of the swarm colony in % of the WT control. (PDF) [file pone.0123646.s002.pdf]

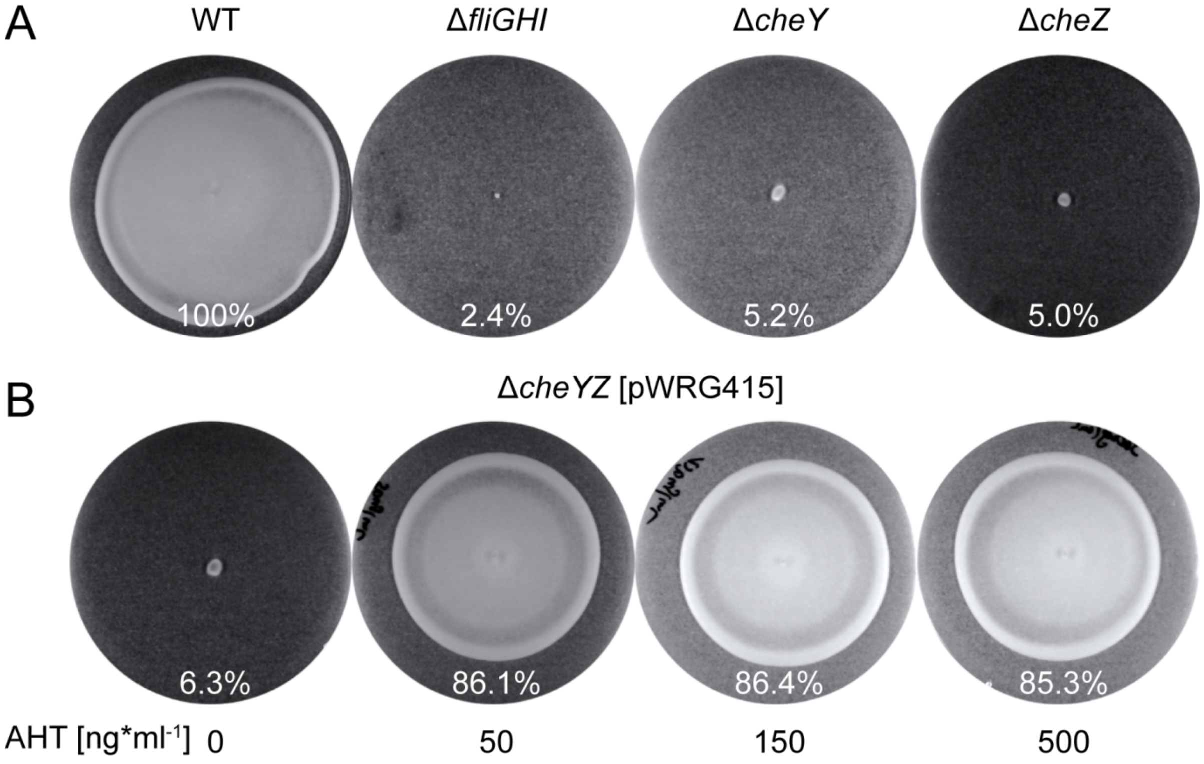

S2 Figure. Functional analysis of CheY-YFP and CheZ-CFP fusion proteins.
Functionality of the chemotaxis system was assessed using the swarming behavior of different *S*. Typhimurium strains on soft agar plates. (A) The swarm colony formation by *Salmonella* NCTC 12023 wild-type (WT) served as a positive control. Strain M913 (Δ*fliGHI*) is a nonmotile control and strains MvP1212 (Δ*cheY*) and WRG105 (Δ*cheZ*) do not show chemotaxis. (B) Swarm colony formation by strain WRG106 Δ*cheYZ* [pWRG415] expressing CheY-YFP and CheZ-CFP upon addition of different concentrations of the inductor anhydrotetracycline (AHT). Percentages indicate the diameter of the swarm colony in % of the WT control.
