# Supplementary material for: A Gateway-Based System for Fast Evaluation of Protein-Protein Interactions in Bacteria
Source: PLoS One. 2015 Apr 9;10(4):e0123646. doi: 10.1371/journal.pone.0123646 (PMC4391838; doi:10.1371/journal.pone.0123646)
Supplement: S1 Table — (DOCX) [file pone.0123646.s003.docx]

**S1 Table. Oligonucleotides used in this study.**

| **Oligonucleotide** | **Sequence (5’→3’), restriction sites underlined** |
| --- | --- |
| Acc65I-SmiI-Pro-tet-for | GGTACCATTTAAATTCGATGGGTGGTTAACTC |
| attB1-entry-n-term-tag-attB4 | GGGGACAAGTTTGTACAAAAAAGCAGGCTGATATCTAATAAGCGGCCGCATAATAAGGATCCTAATAACACCCAACTTTTCTATACAAAGTTGTCCCC |
| attB1-siiF-for | GGGGACAAGTTTGTACAAAAAAGCAGGCTAAGAGGAGGTTACTTATGGATAAAAAACTAGAAC |
| attB3-entry-n-term-tag-attB2 | GGGGACAACTTTGTATAATAAAGTTGGCGATATCTAATAAGCGGCCGCATAATAAGGATCCTAATAATACCCAGCTTTCTTGTACAAAGTGGTCCCC |
| attB3r-rev | GGGGACAACTTTATTATACAAAGTTGTCACGTGTCTCTATC |
| attB3-siiF-for | GGGGACAACTTTGTATAATAAAGTTGAAGAGGAGGTTACTTATGGATAAAAAACTAGAAC |
| attB4r-for | GGGGACAACTTTTCTATACAAAGTTGTCAAAGCTTTAATAAC |
| B1-B4-for | GGGGACAAGTTTGTACAAAA |
| B1‐B4‐rev | GGGGACAACTTTGTATAGAA |
| B3‐B2‐for | GGGGACAACTTTGTATAATA |
| B3‐B2‐rev | GGGGACCACTTTGTACAAGA |
| BglII-L14-HaloTag-for | CAGAGATCTGGCTCTGCGGCGTCTGCGGCGGGCGCGGGCGAAGCGGCGGCGGGATCCGAAATCGGTACTGGC |
| BglII-SplitGLuc1-N-for | GATAGATCTATGAAACCGACCGAAAACAAC |
| BglII-T18-for | GATAGATCTACCATGATTACGCCAAGCTTG |
| BglII-XFP-for | GATAGATCTGTGAGCAAGGGCGAGGAGCTG |
| CheY-Red13-Del-for | gtagtattttATGGCGGATAAAGAGCTTAAATTTTTGGTTattccggggatccgtcgacc |
| CheY-Red13-Del-rev | cgcatccTCACATGCCCAGTTTCTCAAAGATTTTGTTGAGtgtaggctggagctgcttcg |
| CheZ-Del-for | GAACCCGATAAGCGCAGCGCCATCAGGTCAAAAAAGCTTAGTGTAGGCTGGAGCTGCTTC |
| CheZ-Del-rev | AACAAAATCTTTGAGAAACTGGGCATGTGAGGATGCGATGCATATGAATATCCTCCTTAG |
| CmR-ccdB-NotI-rev | CAGGCGGCCGCCAGCATCACCCGACGCACTTTG |
| EcoRV-CmR-ccdB-for | CAGGATATCATGTTCTTCGGGTGATGCTGC |
| ENTR/EXPR-Seq-rev | CAGGAAACAGCTATGACC |
| ENTR-Seq-for | AACGACGGCCAGTCTTAAG |
| E-PCR1-B1-B4 | GGGGACAAGTTTGTACAAAAAAGCAGGCTAAGAGGAGGATATCGCGGCCGCAGGATCCACCCAACTTTTCTATACAAAGTTGTCCCC |
| E-PCR1-B3-B2 | GGGGACAACTTTGTATAATAAAGTTGAAGAGGAGGATATCGCGGCCGCAGGATCCACCCAGCTTTCTTGTACAAAGTGGTCCCC |
| EXPR-Seq-for | TGCTCTACACCTAGCTTCTG |
| HaloTag-NotI-rev | CAGGCGGCCGCCTAACCGGAAATCTCCAGAGTAG |
| HindIII-L14-SnapTag-for | CAGAAGCTTGGCTCTGCGGCGTCTGCGGCGGGCGCGGGCGAAGCGGCGGCGGACAAAGACTGCGAAATGAAGC |
| HindIII-SplitGLuc1-C-for | GATAAGCTTGGTGGCATTGGCGAAGCG |
| HindIII-T25-for | GATAAGCTTATGACCATGATTACGGCTGC |
| HindIII-XFP-for | GATAAGCTTGTGAGCAAGGGCGAGGAGCTG |
| NaeI-RBS-T25-for | CAGGCCGGCAAGAGGAGGAACAGCTATGACCATGCAGCAATC |
| PmlI-RBS-T18-for | CAGCACGTGAAGAGGAGGAACAGCTATGACCATGATTAC |
| Pro-tet-EcoRV-rev | GCGGATATCTTTCTCTATCACTGATAGGGAG |
| siiF-attB2-rev | GGGGACCACTTTGTACAAGAAAGCTGGGTCATTAATAATTTATCCGGAGAAC |
| siiF-attB4-rev | GGGGACAACTTTGTATAGAAAAGTTGGGTCATTAATAATTTATCCGGAGAAC |
| SnapTag-XhoI-rev | CAGCTCGAGCTAACCCAGCCCAGGCTTGCCCAG |
| SplitGLuc1-C-XhoI-rev | ATCCTCGAGTTATTAATCGCCACCCGCAC |
| SplitGLuc1-N-NotI-rev | ATCGCGGCCGCTTATTACTGCGCGCTTTCTTTATCGC |
| T18-NotI-rev | ATCGCGGCCGCTTATTATATCGATTGGCGTTCCAC |
| T18-PmlI-rev | CAGCACGTGGTGGCGTTCCACTGCGCCCAG |
| T25-NaeI-rev | CAGGCCGGCCCTGCAGCCCGCCGCGTGCGCG |
| T25-XhoI-rev | ATCCTCGAGTTATTATATCGATGGTGCAGC |
| XFP-NotI-rev | GATGCGGCCGCTTATTACTTGTACAGCTCGTCCATGC |
| XFP-XhoI-rev | ATCCTCGAGTTATTACTTGTACAGCTCGTCCATGC |
