# Supplementary material for: A Gateway-Based System for Fast Evaluation of Protein-Protein Interactions in Bacteria
Source: PLoS One. 2015 Apr 9;10(4):e0123646. doi: 10.1371/journal.pone.0123646 (PMC4391838; doi:10.1371/journal.pone.0123646)
Supplement: S2 Table — (DOCX) [file pone.0123646.s004.docx]

**S2 Table. Plasmids used in this study.**

| **Plasmid** | **Relevant characteristic(s)** | **Source or Reference** |
| --- | --- | --- |
| p25-N | encodes the T25 fragment (aa 1–224) of CyaA for C-terminal fusions; Km^r^ | [1] |
| pBluescript II SK (+)/ [pSK] | f1 ori; MCS; Ap^r^ | Thermo Scientific (St.-Leon-Rot) |
| pCP20 | FLP expression vector, temperature-sensitive replicon, Ap^r^, Cm^r^ | [2] |
| pDONR™ 221 | *att*P1-; *att*P2-recombination-sites; *ccd*B-Cm^r^-cassette; Km^r^ | Life Technologies |
| pDONR™ 221 P4r-P3r | *att*P4r-; *att*P3r- recombination-sites; *ccd*B-Cm^r^-cassette; Km^r^ | Life Technologies |
| pDONR™ 221 P1-P4 | *att*P1-; *att*P4- recombination-sites; *ccd*B-Cm^r^-cassette; Km^r^ | Life Technologies |
| pDONR™ 221 P3-P2 | *att*P3-; *att*P2- recombination-sites; *ccd*B-Cm^r^-cassette; Km^r^ | Life Technologies |
| pKD13 | *aph* resistance cassette flanked by FRT sites, temperature-sensitive replication (30°C), Km^r^, Ap^r^ | [3] |
| pKD4 | *aph* resistance cassette flanked by FRT sites, temperature-sensitive replication (30°C), Km^r^, Ap^r^ | [3] |
| pKD46 | *araC*-P*_ara_*-*γ*-*β*-*exo* rep101ts arabinose-inducible expression of λ Red-recombinase temperature-sensitive replication (30°C), Ap^r^ | [3] |
| pKNT25-ZIP | leucine zipper of GCN4 fused in frame to T25 fragment; Km^r^ pKT25 derivative | [4] |
| pKT25 | encodes the T25 fragment (aa 1–224) of CyaA for N-terminal fusions; Km^r^ | [4] |
| pMA-GLuc^M43L‑M110L^ | pMA vector with codon-optimized enhanced *Gluc*, Ap^r^ | This work |
| pSCFP3a-C1 | P*_cmv_*-*scfp3a*; Km^r^ | [5] |
| pST98-AS | Low-copy-number vector, I-*Sce*I endonuclease under control of tetracycline-inducible promoter (P*tetA*), Ap^r^ | [6] |
| pSYP2-C1 | P*_cmv_-syfp2*; Km^r^ | [5] |
| pUT18 | T18 fragment (aa 225–399) of CyaA for C-terminal fusions, Ap^r^ | [4] |
| pUT18C | T18 fragment (aa 225–399) of CyaA for N-terminal fusions, Ap^r^ | [4] |
| pUT18C-ZIP | leucine zipper of GCN4 fused in frame T18 fragment, Ap^r^ pUT18C derivative | [4] |
| pWRG175 | derivative of pDONR™ 221 P1-P4 with EcoRV and NotI restriction enzyme recognition sites deleted | This work |
| pWRG176 | derivative of pDONR™ 221 P3-P2 with EcoRV and NotI restriction enzyme recognition sites deleted | This work |
| pWRG179 | *att*L1-RBS*-*MCS*-att*L4; Km^r^ | This work |
| pWRG180 | *att*L3-RBS*-*MCS*-att*L2; Km^r^ | This work |
| pWRG201 | pSK-vector with dual Gateway-construct; Ap^r^ | This work |
| pWRG239 | pSK-vector with P*_tetA_* and dual Gateway-construct; Ap^r^ | This work |
| pWRG243 | *att*L3*-*RBS*-scfp3a-att*L2 | This work |
| pWRG256 | *att*L3-RBS*-cheY-att*L2; Km^r^ | This work |
| pWRG257 | *att*L1-RBS*-cheZ-att*L4; Km^r^ | This work |
| pWRG258 | *att*R4-*att*R3-recombination-sites, *rrn*B-terminator; P*_tetA_*; Km^r^ | This work |
| pWRG367 | *att*L1-RBS*-invB-att*L4; Km^r^ | This work |
| pWRG368 | *att*L3-RBS*-sipA-att*L2; Km^r^ | This work |
| pWRG369 | *att*L3-RBS*-sipA*^48-685^*-att*L2; Km^r^ | This work |
| pWRG392 | pWSK29 with P*_tetA_* and dual Gateway-construct; Ap^r^ | This work |
| pWRG415 | P*_tetA_-cheZ*-*scfp3a-rrnB*-terminator-P*_tetA_-cheY-syfp2;* Ap^r^ | This work |
| pWRG438 | *att*L1*-*RBS*-* C‑*gluc*^M110L^_106-185_*-att*L4 | This work |
| pWRG439 | *att*L3*-*RBS*-* N*-gluc*^M43L^_M18-105_*-att*L2 | This work |
| pWRG448 | P*_tetA_-invB*-*t25*-*rrn*B-Terminator-P*_tetA_-sipA*-*t18*; Ap^r^ | This work |
| pWRG450 | P*_tetA_-invB*-*t25*-*rrn*B-Terminator-P*_tetA_-sipA*_48-685_-*t18*; Ap^r^ | This work |
| pWRG467 | *att*L1-RBS*-leucine-zipper-att*L4; Km^r^ | This work |
| pWRG468 | *att*L3-RBS*-leucine-zipper-att*L2; Km^r^ | This work |
| pWRG469 | P*_tetA_*-C-gluc^M110L^_106-185_-*rrnB*-terminator-P*_tetA_*-N-gluc^M43L^_M18-105_; Ap^r^ | This work |
| pWRG471 | P*_tetA_*-*invB*-C-gluc^M110L^_106-185_-*rrnB*-terminator-P*_tetA_*-*sipA*-N-gluc^M43L^_M18-105_; Ap^r^ | This work |
| pWRG473 | P*_tetA_*-*invB*-C-gluc^M110L^_106-185_-*rrnB*-terminator-P*_tetA_*-*sipA*^48-685^-N-gluc^M43L^_M18-105_; Ap^r^ | This work |
| pWRG482 | P*_tetA_-leucine-zipper-t25*-*rrn*B-Terminator-P*_tetA_-leucine-zipper-t18;* Ap^r^ | This work |
| pWRG506 | *att*L1-RBS*-t18-att*L4; Km^r^ | This work |
| pWRG507 | *att*L3-RBS*-t25-att*L2; Km^r^ | This work |
| pWRG512 | P*_tetA_*-*att*R1-*ccd*B-Cm^r^-*att*R2 | This work |
| pWRG534 | P*_tetA_-t25*-*rrn*B-Terminator-P*_tetA_-t18*; Ap^r^ | This work |
| pWRG575 | P*_tetA_*-*scfp*3a-*att*R1-*ccdB*-CmR-*att*R2 | This work |
| pWRG583 | *att*L1-EcoRV-NotI-TAATAA-BamHI-TAATAA-*att*L4 | This work |
| pWRG584 | *att*L3-EcoRV-NotI-TAATAA-BamHI-TAATAA-*att*L2 | This work |
| pWRG597 | *att*L1-*leucine zipper*-TAATAA-*att*L4 | This work |
| pWRG598 | *att*L3-*leucine zipper*-TAATAA-*att*L2 | This work |
| pWRG602 | P*_tetA_-cheZ*-*snap-tag-rrnB*-terminator-P*_tetA_-cheY-halotag;* Ap^r^ | This work |
| pWRG623 | P*_tetA_-t25*-*leucine-zipper*-*rrn*B-Terminator-P*_tetA_-t18-leucine-zipper*; Ap^r^ | This work |
| pWRG-B2H-DEST-C | P*_tetA_*-*att*R1-*ccd*B-Cm^r^-*att*R2-*t18* | This work |
| pWRG-B2H-DEST-N | *P_tetA_-RBS-t25-attR1-ccdB-Cm^r^-attR2-t18* | This work |
| pWRG-B2H-ENTR-C | *att*R4-*att*R3-rekombination-sites, *rrn*B-terminator; P*_tetA_*, *t25*; Km^r^ | This work |
| pWRG-B2H-ENTR-N | *att*R4-*t25*-*rrn*B*-*Terminator-P*_tetA_-*RBS*-t18*-*att*R3 | This work |
| pWRG-ENTR-C1 | *att*L1-RBS*-*EcoRV-*ccd*B-Cm^r^-BglII-NotI*-att*L4 | This work |
| pWRG-ENTR-C2 | *att*L3-RBS-EcoRV-*ccd*B-Cm^r^-BglII-NotI-*att*L2 | This work |
| pWRG-ENTR-N1 | *att*L1-EcoRV-*ccd*B-Cm^r^-NotI-TAATAA-BamHI-TAATAA-*att*L4 | This work |
| pWRG-ENTR-N2 | *att*L3-EcoRV-*ccd*B-Cm^r^-NotI-TAATAA-BamHI-TAATAA-*att*L2 | This work |
| pWRG-FRET-DEST-C | P*_tetA_*-*att*R1-*ccd*B-Cm^r^-*att*R2-*syfp2* | This work |
| pWRG-FRET-ENTR-C | *att*R4-*att*R3-recombination-sites, *rrn*B-terminator; P*_tetA_*; *scfp3a*; Km^r^ | This work |
| pWRG-GLUC-DEST-C | P*_tetA_*-*att*R1-*ccd*B-Cm^r^-*att*R2-N*-gluc*^M43L^_M18-105_ | This work |
| pWRG-GLUC-ENTR-C | *att*R4-*att*R3-recombination-sites, *rrn*B-terminator, P*_tetA_*, C‑*gluc*^M110L^_106-185_; Km^r^ | This work |
| pWRG-HALO-DEST-C | P*_tetA_*-*att*R1-*ccd*B-Cm^r^-*att*R2-*halotag*; Ap^r^ | This work |
| pWRG-SNAP-ENTR-C | *att*R4-*att*R3-recombination-sites, *rrn*B-terminator; P*_tetA_*; *snap-tag*; Km^r^ | This work |
| pWSK29 | Low copy-number vector, Ap^r^ | [7] |

**Supplementary Literature**

1. Claessen D, Emmins R, Hamoen LW, Daniel RA, Errington J, Edwards DH. Control of the cell elongation-division cycle by shuttling of PBP1 protein in *Bacillus subtilis*. Molecular microbiology. 2008;68(4):1029-46. doi: 10.1111/j.1365-2958.2008.06210.x. PubMed PMID: 18363795.

2. Cherepanov PP, Wackernagel W. Gene disruption in *Escherichia coli*: Tc^R^ and Km^R^ cassettes with the option of Flp-catalyzed excision of the antibiotic-resistance determinant. Gene. 1995;158(1):9-14. PubMed PMID: 7789817.

3. Datsenko KA, Wanner BL. One-step inactivation of chromosomal genes in *Escherichia coli* K-12 using PCR products. Proceedings of the National Academy of Sciences of the United States of America. 2000;97(12):6640-5. PubMed PMID: 10829079.

4. Karimova G, Pidoux J, Ullmann A, Ladant D. A bacterial two-hybrid system based on a reconstituted signal transduction pathway. Proceedings of the National Academy of Sciences of the United States of America. 1998;95(10):5752-6. Epub 1998/05/20. PubMed PMID: 9576956.

5. Kremers GJ, Goedhart J, van Munster EB, Gadella TW, Jr. Cyan and yellow super fluorescent proteins with improved brightness, protein folding, and FRET Forster radius. Biochemistry. 2006;45(21):6570-80. Epub 2006/05/24. doi: 10.1021/bi0516273. PubMed PMID: 16716067.

6. Pósfai G, Kolisnychenko V, Bereczki Z, Blattner FR. Markerless gene replacement in *Escherichia coli* stimulated by a double-strand break in the chromosome. Nucleic acids research. 1999;27(22):4409-15. PubMed PMID: 10536150.

7. Wang RF, Kushner SR. Construction of versatile low-copy-number vectors for cloning, sequencing and gene expression in *Escherichia coli*. Gene. 1991;100:195-9. PubMed PMID: 2055470.
