# Supplementary material for: A Gateway-Based System for Fast Evaluation of Protein-Protein Interactions in Bacteria
Source: PLoS One. 2015 Apr 9;10(4):e0123646. doi: 10.1371/journal.pone.0123646 (PMC4391838; doi:10.1371/journal.pone.0123646)
Supplement: S3 Table — (DOCX) [file pone.0123646.s005.docx]

S3 Table. Efficiencies of LR reactions.

| **Destination vector (reporter gene/fragment), 3x Entry clone (insert size)** | **Ap^r^ CFUs** | **Clones screened** | **Correct clones** |
| --- | --- | --- | --- |
| Expression clone: pWRG534 | | | |
| pWRG512 (no reporter gene) | 28 | ^a^16, ^b^7 | ^a^7, ^b^6 |
| pWRG506 *att*L1*-*RBS*-t25-att*L4 (788 bp) |  |  |  |
| pWRG507 *att*L3-RBS-*t18*-*att*L2 (641 bp) |  |  |  |
| pWRG258 *att*R4-*rrnb-*Terminator-P*_tetA_*- *att*R3 (242 bp) |  |  |  |
| Expression clone: pWRG448 | | | |
| pWRG-B2H-DEST-C (*t*18*-*C) | 16 | ^a^8, ^b^3 | ^a^4, ^b^3 |
| pWRG367 *att*L1*-*RBS*-invB-att*L4 (425 bp) |  |  |  |
| pWRG368 *att*L3-RBS-*sipA*-*att*L2 (2075 bp) |  |  |  |
| pWRG-B2H-ENTR-C *att*R4-*t25-rrnb-*Terminator-P*_tetA_*- *att*R3 (1018 bp) |  |  |  |
| Expression clone: pWRG450 | | | |
| pWRG-B2H-DEST-C (*t*18*-*C) | 12 | ^a^8, ^b^4 | ^a^3, ^b^3 |
| pWRG367 *att*L1*-*RBS*-invB-att*L4 (425 bp) |  |  |  |
| pWRG369 *att*L3-RBS-*sipA*_48-685_-*att*L2 (1937 bp) |  |  |  |
| pWRG-B2H-ENTR-C *att*R4-*t25-rrnb-*Terminator-P*_tetA_*- *att*R3 (1018 bp) |  |  |  |
| Expression clone: pWRG482 | | | |
| pWRG-B2H-DEST-C (*t*18*-*C) | 48 | ^a^8, ^b^4 | ^a^7, ^b^4 |
| pWRG467 *att*L1*-*RBS*-leucine-zipper-att*L4 (149 bp) |  |  |  |
| pWRG468 *att*L3-RBS-*leucine-zipper*-*att*L2 (149 bp) |  |  |  |
| pWRG-B2H-ENTR-C *att*R4-*t25-rrnb-*Terminator-P*_tetA_*-*att*R3 (1018 bp) |  |  |  |
| Expression clone: pWRG623 | | | |
| pWRG-B2H-DEST-N (N-*t*25) | 9 | ^a^3, ^b^3 | ^a^3, ^b^3 |
| pWRG597 *att*L1*-leucine-zipper*-TAATAA-*att*L4 (149 bp) |  |  |  |
| pWRG598 *att*L3*-leucine-zipper*-TAATAA-*att*L2 (149 bp) |  |  |  |
| pWRG-B2H-ENTR-N *att*R4*-t25-rrnb-*Terminator-P*_tetA_*-RBS-*t18-att*R3 (1598 bp) |  |  |  |
| Expression clone: pWRG471 | | | |
| pWRG-GLUC-DEST-C (Gluc_105_) | 10 | ^a^8, ^b^4 | ^a^4, ^b^4 |
| pWRG367 *att*L1 RBS*-invB-att*L4 (425 bp) |  |  |  |
| pWRG368 *att*L3-RBS-*sipA*-*att*L2 (2075 bp) |  |  |  |
| pWRG-GLUC-ENTR-C *att*R4-C-*gluc*_106_*-rrnb-*Terminator-P*_tetA_*-*att*R3 (502 bp) |  |  |  |
| Expression clone: pWRG473 | | | |
| pWRG-GLUC-DEST-C (Gluc_105_) | 9 | ^a^8, ^b^4 | ^a^3, ^b^3 |
| pWRG367 *att*L1 RBS*-invB-att*L4 (425 bp) |  |  |  |
| pWRG369 *att*L3-RBS-*sipA*_48-685_-*att*L2 (1937 bp) |  |  |  |
| pWRG-GLUC-ENTR-C *att*R4-C-*gluc*_106_*-rrnb-*Terminator-P*_tetA_*-*att*R3 (502 bp) |  |  |  |
| Expression clone: pWRG469 | | | |
| pWRG512 (no reporter gene) | 19 | ^a^8, ^b^4 | ^a^6, ^b^4 |
| pWRG438 *att*L1 RBS*-*C-*gluc*_106_*-att*L4 (269 bp) |  |  |  |
| pWRG439 *att*L3-RBS*-*N-*gluc*_105_-*att*L2 (293 bp) |  |  |  |
| pWRG258 *att*R4*-rrnb-*Terminator-P*_tetA_*-*att*R3 (242 bp) |  |  |  |
| Expression clone: pWRG415 | | | |
| pWRG-FRET-DEST-C (*syfp*2-C) | 183 | ^a^8, ^b^4 | ^a^7, ^b^4 |
| pWRG257 *att*L1*-*RBS*-cheZ-att*L4 (662 bp) |  |  |  |
| pWRG256 *att*L3-RBS-*cheY*-*att*L2 (407 bp) |  |  |  |
| pWRG-FRET-ENTR-C *att*R4-*scfp3a-rrnb-*Terminator-P*_tetA_*-*att*R3 (976 bp) |  |  |  |
| Expression clone: pWRG602 | | | |
| pWRG-HALO-DEST-C (Snap-tag-C) | 32 | ^a^8, ^b^4 | ^a^6, ^b^4 |
| pWRG257 *att*L1*-*RBS*-cheZ-att*L4 (662 bp) |  |  |  |
| pWRG256 *att*L3-RBS-*cheY*-*att*L2 (407 bp) |  |  |  |
| pWRG-SNAP-ENTR-C *att*R4-*halotag-rrnb-*Terminator-P*_tetA_*-*att*R3 (976 bp) |  |  |  |

^a^colony PCR; ^b^control digest
